# Factors Associated with Knowledge, Attitude and Practices of Common Eye Diseases in General Population: A Multicenter Cross-Sectional Study from Pakistan

**DOI:** 10.3390/ijerph16091568

**Published:** 2019-05-05

**Authors:** Mingyue Zhao, Ali Hassan Gillani, Fakir Mohammad Amirul Islam, Wenjing Ji, Khezar Hayat, Zongjie Li, Jamshaid Akbar, Abu Bakar Ahmed, Ans Azam, Imran Masood, Yu Fang

**Affiliations:** 1 Department of Pharmacy Administration and Clinical Pharmacy, School of Pharmacy, Xi’an Jiaotong University, Xi’an 710061, China; mingyue0204@xjtu.edu.cn (M.Z.); hassangillaniali@yahoo.com (A.H.G.); yfyx_8312@163.com (W.J.); khezar.hayat@uvas.edu.pk (K.H.); zongjieli@yahoo.com (Z.L.); 2 Center for Drug Safety and Policy Research, Xi’an Jiaotong University, Xi’an 710061, China; 3 Shaanxi Centre for Health Reform and Development Research, Xi’an 710061, China; 4 Department of Statistics, Data Science and Epidemiology, Faculty of Health, Arts and Design, Swinburne University of Technology, Hawthorn VIC 3122, Australia; fislam@swin.edu.au; 5 Organization for Rural Community Development, Dariapur, Narail 7500, Bangladesh; 6 Institute of Pharmaceutical Sciences, University of Veterinary and Animal Sciences, Lahore 54000, Pakistan; 7 Department of pharmaceutical Sciences, The Superior College, Lahore, Punjab 54000, Pakistan; dr.jamshaidsheikh@gmail.com; 8 Department of Pharmacy, Islamia University of Bahawalpur 63100, Pakistan; abubakarpharmacist14@gmail.com (A.B.A.); ans.a.goheer@gmail.com (A.A.); drimranmasood@gmail.com (I.M.)

**Keywords:** knowledge, attitude, practice, eye disease, Punjab Pakistan

## Abstract

High prevalence of blindness coupled with low public awareness of eye diseases is a severe problem in Pakistan. The objective of this study was to evaluate the awareness, attitude and practices related to common ocular problems and eye care use among general population of Punjab, Pakistan. Data were collected cross-sectionally, using random selection of participants from five districts of Punjab, Pakistan. A pre-tested questionnaire was used to collect the data from January to March 2017. The ordinal data were converted to interval--based data by Rasch analysis, which is termed as “knowledge score” and ranges from −3 to +3. Descriptive statistics, linear regression, binary logistic regression and chi square analysis were performed to evaluate the data. Of the 2073 people contacted, 96.4% (2019) responded the interviewer. The mean (SD) age was 39.9 (11.4) years, majority were males (64.8%) and only 3.1% were illiterate. The majority (68.1%) of respondents were aware that blindness can be prevented and are least aware of age-related macular degradation (31.4%). Multivariate analysis to knowledge scores demonstrated that male gender (β = 0.53 95%CI = 0.39, 0.66, *p* < 0.001), higher education (β = 0.31 95%CI = 0.25, 0.37, *p* < 0.001) were significantly associated with knowledge score. Eighty-two percent of our respondents had a positive attitude towards health-seeking behavior and those who had adequate income on most of occasions were 1.82 (AOR = 1.82 95%CI = 1.26, 2.62 *p* = 0.001) times more prone towards health-seeking behavior than those who had insufficient income. Out of all respondents, 21.5% had an eye examination at least once a year and this frequency was significantly higher in lower age, females, high education and high Socio Economic Status level (*p* < 0.05). Among the general population of Pakistan, awareness about prevention of blindness and refractive error was optimum, while awareness of blindness causing eye conditions, like age-related macular degradation was low. Intensive improvements in the health literacy and public interventions—specifically in old age people, males and respondents with lower education—are urgently needed.

## 1. Introduction

Global estimates report that 191 million population have moderate to extreme vision impairment (MEVI), and among them 32.4 million are blind, with a high proportion of females (60%) [1]. Although South Asian countries encounter a high burden of age-related blindness, this is still lower than Sub Saharan countries (4.4% vs. 6.0%), whereas prevalence of the MEVI is slightly higher in South Asian countries (23.6%) than in Oceana (18.9%) [1]. Uncontrolled refractive errors (43%) are the foremost reason for visual impairment (VI) and are trailed by cataracts (33%), diabetic retinopathy (DR), glaucoma and age-related macular degradation (ARMD) [2]. Similarly, a study in Iran revealed that almost 32 out of 44 persons with blinding issues had cataracts, 10 out of 44 had diabetic retinopathy and more than two out of 44 had glaucoma [3]. Additionally, other studies from Iran provided evidence of poor eye examination as only 22% of diabetic patient had had eye checkups and two out of five non-diabetic individuals with eye disease had a history of proper eye checkups [4,5]. The large-scale population-based survey in Pakistan in 2006 reported that prevalence of blindness was 2.7% and was highest in Punjab and Baluchistan and was severe in the rural areas, while it was lowest in North West Frontier Province (known as Khyber Pakhoonkhwa) [6]. Similarly, the study by Dineen et al., in 2007 articulated that among the many causes of blindness the cataract (51.5%) is the foremost cause of blindness in Pakistan, followed by corneal opacity (11.8%), and glaucoma (7.1%) [7].

Knowledge regarding the ARMD in Hong Kong Chinese population (9.2%) [8] and related to glaucoma in Iranian population (46.6%) is really low [9]. The level of public awareness related to major blindness causes has been reported to be low even in developed countries [10,11]. However, health promotion helps to reduce the encumbrance of eye ailments and ultimately limit avoidable causes of blindness [12] as many studies revealed the importance of blood pressure and glycemic control in the development and progression of diabetic retinopathy reduces the numbers of cases [13]. From the past evidences of knowledge, attitude and practice (KAP) studies it is obvious that immense need for the knowledge regarding risk factor, control, prevention diagnosis and management subsists among the community [14,15]. The need of the hour is to alleviate the diseases burden by proper implementation and reinforcement of public health policies specifically focusing on VI risk factors. Factors associated with the public health plans are scarcity of awareness of eye diseases [16,17] that is coupled with poorer prevention [18], inappropriate eye care [19] and inadequate treatment [20].

Very few studies have been performed in Pakistan to evaluate the KAP ofblindness. The general public is never being interviewed about the KAP of eye disease, except only one study by Memon assessing the KAP regarding diabetes and diabetic retinopathy in Karachi [21], which evaluated the knowledge of 271 respondents regarding diabetes and DR, and only focused on DR while all other eye diseases were not the subjects of interviews. Therefore, in order to construct the interventional and prevention policies the KAP of general population should be measured so it will be helpful to estimate the awareness gaps in general population which should be covered. Therefore, in our study we report the KAP and associated factors in terms of demographics in area of Punjab, Pakistan.

## 2. Materials and Methods

### 2.1. Study Area

Pakistan is constituted of 5 provinces and these provinces are divided into the sub-levels of division level, districts level and Tehsils level. Townships or villages are the lowest class of socio-demographic status. Almost one fourth (26%) of the total area of Pakistan is taken by Punjab, which is divided into 9 divisions and 36 districts, which holds 60% of the population of Pakistan [22,23]. Among these 36 districts, Bahawalnagar, Rahim Yar khan, Multan, Sialkot and Faisalabad were randomly selected. Each district headquarter city and rural community was selected randomly.

### 2.2. Study Population and Recruitment

Individuals aged over 18, regardless of religion, sect, sex and socioeconomic status (SES) and who were willing to take part in activity, were interviewed. The survey was conducted by door-to-door visits and the data collector conducted interviews of willing participants. Houses were selected randomly in the cities and similarly in selected villages. Only one individual was selected from each house based on selection by lottery method [24]. Cross-sectional data collection was carried out from January to March 2017.

### 2.3. Statement of Ethical Approval

This research activity was approved by Ethics Committee for Medical Research of Xi’an Jiaotong University Shaanxi, China (Ethical approval # ED-12-2018). At the same time, it was also approved by the ethics committee of Islamia University Bahawalpur. Written approval was taken from literate population who were able to read and sign the consent form. In other cases, the data collector signed the consent form after seeking permission from illiterate persons who were unable to understand the consent form. Participants were made completely aware about the option to leave the study at any stage.

### 2.4. KAP Questionnaire

A comprehensive questionnaire was constructed to gather data regarding the socio-demographics and to evaluate KAP of the general population. The awareness was assessed by simply asking the participants whether they had heard of a particular eye disease (9 items), attitude was estimated by asking the views of respondents toward seeking eye treatment if they have an eye problem (1 item), and practice was assessed by evaluating the frequency of the eye checks (1 item). These items for assessing the KAP of eye disease were tailored from the instrument used in Cambodia [25] and Bangladesh [26]. The primary questionnaire was in the English language, and was translated into Urdu with the help of senior educator. Each individual item was translated into Urdu under supervision of both the senior educator and the principal investigator (PI). After being agreed upon, the final Urdu version of the questionnaire was used for data collection. For instance, Cataract is known as Motia Bund in Urdu, so to aid understanding Motia Bund was used and similarly with all other items. The nature of attitude was evaluated by asking “will they seek treatment for eye disease”, and attitude was documented as positive if the answer is yes. Questions relating to knowledge and attitudes were in binary format (yes/no) while questions related to practice measure frequency of eye check and were listed as follows: 1: more than once a year, 2: at least once in a year 3: whenever you have eye disease and 4: never. Age was categorized as 18–30, 31–45, 46–60, and more than 60, and similarly education was categorized as Nil, 1–5 (up to primary), 6–10 (primary to metric), 10–12 (up to intermediate) and more than intermediate. Socioeconomic status (SES) was evaluated using the method of Cheung et al. [27] and Islam et al. [26] as the income of majority of the population was not taxable. SES was evaluated with the question “How would you like to classify your SES status over the last twelve months, in regard of household food utilizations?”. The answers were categorized as (i) inadequate resources for the complete year, (ii) inadequate resources for few times, (iii) balance, and (iv) adequate resources most of the time.

The questionnaire was further checked for validity and consistency in our study population. Before finalizing the questionnaire, a pilot test was done in selected areas among 30 individuals from each district. The individuals were asked to assessment wording, appropriateness, and comprehension of the questionnaire. The data of the pretest were not included in the final analysis.

### 2.5. Statistical Analysis

Descriptive statistics with respective percentages were used to describe all demographic variables including age, gender, locality, SES, education and occupation status. Rasch analysis technique was employed to transform categorical or ordinal data into the interval-based measures [26]. The Rasch model, named after the Danish mathematician Rasch [28], shows what can be expected of responses to items if measurement is to be achieved [29]. We used it to transform person knowledge item measures into logarithmic values expressed as “knowledge scores”. The categorization of the score was between −3 to +3. Those scored in negative values indicated knowledge below average, those scored 0 were categorized as having average knowledge and those who scored in positive value were categorized as having an above-average score. The outcome of the knowledge score in logarithmic scale was associated with demographics using linear regression. Beta coefficient with respective 95%CI was calculated after adjusting for gender, age, SES and education. Chi square analysis used to demonstrate the significant difference of each knowledge response with demographics (age, gender, locality, SES and education). Attitude outcome was binary, so the association between the variables was done by using binary logistic regression (AOR, 95%CI). Chi square test was used to illustrate the considerable difference between the practice items. Complete statistical evaluation was conducted using SPSS version 18 (SPSS Inc., Chicago, IL, USA).

## 3. Results

Of the 2073 people approached, 2019 responded to the study (response rate 96.4%) and the mean age ± SD was 39.92 ± 11.4. Of the 2019, 64.8% were males. Rural population was slightly higher than urban population (51.5% vs 48.5%), 3.1% were illiterate and 36.7% had education level higher than intermediate. More than half of the population (55.6%) had balanced resources while 11.0% had insufficient income for most of the time. (Table 1)

### 3.1. Knowledge Assessment

**Figure 1:**
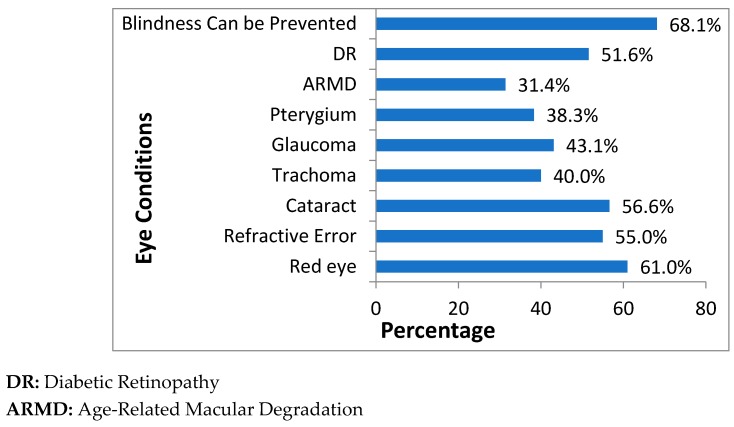
Illustrates the Knowledge level of each eye disease status.

The positive responses to the knowledge questions had significant variations and varied from lowest 31.4%, i.e., “have you ever heard of age-related macular degradation (ARMD)?” to highest 68.1%, i.e., “do you know that blindness can be prevented?” Among all respondents, 61.0% had heard of red eye, 56.6% were aware of cataract, 55% were aware of trachoma and 51.6% heard of the DR. Knowledge about trachoma, pterygium and glaucoma was suboptimal and only 40.0%, 38.3% and 43.1% had heard of these diseases, respectively (Figure 1). Gender, age groups and education had significant differences with almost all the eye disease items, while only age had insignificant association with the awareness of DR, e.g., 47.5% of the population in the age group 31–45 were aware of DR, which is same as the population in age group 46–60 (53.3%). In terms of locality, awareness of red eye (rural 67.1% vs urban 54.5%), cataract (rural 54% vs urban 59.5%) and ARMD (rural 29.4% vs urban 33.6%) was significantly different. In the case of SES, insignificant association was apparent only in the awareness of the cataract, i.e., 59.3% of respondents who have adequate resources for the most of time were aware of cataract, which is trivially different from the respondents who have adequate balance for most of year (57.7%) (Table 2 and Table 3).

### 3.2. Demographic Association with Knowledge Score Using Rasch Analysis.

The mean (95%CI) of the knowledge score was −0.161 (−0.0819, 0.0498) whereas 48.6% (981/2019) had knowledge score in the optimum range, i.e., log value ≥ 0. After adjustment of variables in multivariate model, significant higher scores were observed in males as compared to females, β (95%CI) 0.53 (0.39, 0.66), *p* < 0.001) and increment in every education level was also associated with increment in “knowledge score” as compared to no education, 0.31 (0.25, 0.37), *p* < 0.001. Similarly, those from urban areas had significantly higher “knowledge score”, as compared to rural residents, 0.21 (0.09, 0.34), *p* = 0.001 (Table 4).

### 3.3. Attitudes

The general attitude towards seeking eye treatment was positive. Eighty-two percent out of the total were agreeable to seeking treatment for eye disease. In terms of binary regression results the outcome was insignificantly related to gender and residence. Individuals with the knowledge higher than the intermediate were insignificantly less knowledgeable as compared to those who had no education (AOR = 0.46 95% CI 0.16, 1.33 *p* = 0.152). The respondents who had adequate income for most of the year possessed 1.82 times higher positive attitude than those who had insufficient balance for most of the time (AOR = 1.82 95%CI 1.26, 2.62, *p* = 0.001) (Table 5).

### 3.4. Practice

Twenty-four percent of our population had never gone for an eye checkup, 37.3% reported that they did have an eye examination when they had some eye problem, 21.5% had an eye examination once in the previous year and only 17.2% reported having an eye checkup more than once a year. Population in the range 18–30 were more keen to have an eye examination more than once a year (21.2%) than those older than 60 (8.5%). For those who were more literate than intermediate the frequency of eye check was high (22.5%) as compared to people of no education (17.7%). None of the demographic variable was in insignificant relation with frequency (*p* < 0.05). Self-reporting of study population revealed that 824 (40.8%) respondents had history of some kind of eye problem. Among those who had some history of eye disease, 24.5% had an eye check at least once a year, which is considerably higher than those who were not diagnosed with any disease (19.5% did go for an eye check at least once in a year) (Table 6). 

## 4. Discussion

Our study is the first of its kind, which evaluated the KAP of common eye ailments from the general population of Pakistan. The major conclusion of our study was that awareness about common eye conditions among the Pakistani population was poor. More than two thirds (68.5%) of the population were unaware of ARMD and deficiency was more prominent in old people, rural community and uneducated people. The outcome of this study is two-fold. Firstly, this study showed insignificant adequacy regarding knowledge and awareness about the common eye conditions among the general public which is more intense in population with rural locality, low SES, and low educational level. Secondly, the community with low education, low SES and rural locality were less likely to have regular eye checks. This suggests that increase in health literacy will ultimately improve health-seeking behavior surrounding eye conditions. Like some developing countries such as Bangladesh [26], tertiary health care facilities are providing appropriate ophthalmic services in Pakistan. It was beyond this project to demonstrate the prevalence of different eye diseases in our population, but evidence suggested that increasing knowledge and understanding correspondingly increased the regular eye examinations which definitely reduce the ocular diseases and reduce economic burden due to ocular disturbances [30].

Our study results were dissimilar with the results from a study in Bangladesh. The lowest awareness, in our study, was seen about the ARMD which is 31.4% to highest awareness about the fact that blindness can be prevented (68.1%) whereas, in the study by Islam et al. the minimum awareness was seen for DR (4%) and maximum for cataracts (91%), although adequate awareness in both the studies was low and is associated with education status [26]. Similarly, SES and educational status were associated with the increase in awareness of diabetes in Bangladesh [31]. Low ocular disease awareness was seen among the population with low education and low SES in developing [30,32] as well as in developed countries [33]. Slightly higher than half (55%) of our respondents showed good knowledge towards refractive error, which is in accordance with the study by Alemayehu et al., which showed that 55.9% of participants had good knowledge towards refractive error [34]. In this study, awareness related to cataract and DR is 56.6% and 51.6%, respectively. These results are not in accordance with the results of the study by Marzieh et al., which showed 82.9% were aware of cataracts and 86.2% were aware of DR [9]. Additionally, results regarding the cataract are dissimilar with the study of Lau et al., which showed awareness of 90% respondents [8]. Another interesting finding is that the entire knowledge items were significantly associated with gender (females were significantly higher in awareness than males) which contradicts the results of a study in Bangladesh in which insignificant difference in awareness of almost all the items exists [26]. Similarly, our results contradict the results of another study by Islam et al., where a significantly higher level of awareness about diabetes and its risk factors was seen among the males [31]. However, these comparisons cannot be considered as true comparisons as the studies by Islam et al., which were conducted only in rural communities, as compared to our population which has both rural and urban population. However, these are close comparisons as almost half of our population is from rural areas with equivalent SES and educational status as in Bangladesh.

In terms of association of an individual disease item with the demographics, in our study population over the age of 60 were 1.21 times (AOR = 1.21 95%CI = 0.56, 2.61) more aware of trachoma as compared to population in age group 18–30. These outcomes were out of line with the previous findings of Dandona, in which odds of being aware increase with age, i.e., population in age group 60–69 were 3.25 times (AOR = 3.25 95%CI = 2.10–5.0) more aware of cataract than population in age group 16–29 [30]. In case of glaucoma and DR, our study has provided evidence that there is increase in awareness with the increase of education level, which is also apparent in the previous studies [8,30]. Inequity subsists in awareness intensity among the males and females. In current study, the females were 1.54 times (AOR = 1.54 95%CI = 1.25, 1.88) more aware for cataract, 1.68 times (AOR = 1.68 95%CI = 1.37, 2.07) more for trachoma and 1.51 times (AOR=1.51 95%CI = 1.23, 1.84) more aware for glaucoma. These results are in accordance with the previous study in Iran where females were 1.48 times more (AOR 1.48 95% CI 1.06, 2.06) aware about cataract and 1.93 times more aware about glaucoma (AOR 1.93 95%CI 1.13, 3.32) [9] but are absolutely contrary to the findings of study in Nepal where males were 1.77 times (OR = 1.77 95% CI = 1.46–2.14), 1.38 times (OR = 1.38 95% CI = 1.09, 1.75) and 1.67 times (OR = 1.67 95%CI = 1.10–2.52) more aware about cataract, glaucoma and trachoma, respectively [32].

Attitude concerning treatment of ocular diseases among our cohort was remarkably high and 82% of our population was of the view that they would go to a physician to seek treatment upon noticing any eye ailment. These results were in accordance with the previous study of Islam et al., which showed that 90% of the population was willing to accept medical care for ocular disturbances [26]. Despite the fact, it is apparent from the previous finding that attending eye examinations is a challenge for the general population in developing countries [26]. However, evidence also signifies that alleviating the scarcity of knowledge and awareness ultimately reduces the delay in diagnosis among rheumatoid arthritis patients in UAE [33] and augments the involvement of participants in screening of breast cancer in Hawaii [35].

Practice regarding the eye examination among our cohort was positive, and only 24% of our population had never had an eye examination, while 17.2% had an eye check more than once in a year. Practice of our population is far better than that seen in the study by Paudel et al., in which 53.5% of respondents had never had an eye check [36]. In the present study, frequency of at least one visit to the doctor was considerably high among those who had eye disease (24.5%) than those who did not have eye disease (19.5%). These results coincide with a study on Bangladesh [26]. Similarly, results from developed countries like Australia illustrate good health-seeking practices and 61% of the participants visited either an optometrist or ophthalmologist, at least once in the past two years [11]. Studies related to DR in Low Resource Nations indicated the poor practice and behavior of patients toward eye checks. Those patients are motivated to visit a doctor if their vision is threatened or there is an abrupt disturbance in vision [37].

### Limitations

Our study had some strengths and drawbacks. In terms of strengths, to begin with, our study consisted of face-to-face interviews by trained data collectors that ensure completeness and validity of data collection. Furthermore, face-to-face interviews remove ambiguities among the general population and are helpful in describing the purposes of research activities. Secondly, a sophisticated and reliable technique (Rasch analysis) was adopted to analyze the data. In terms of drawbacks, first we selected the cities randomly and similarly village in selected districts were targeted randomly. However, efforts were made to collect the data from different geographic region of Punjab. Second, the sample mainly consists of male students which may indicate a bias in the random selection of the respondents. Our response to this is that we did not randomly select participants from multiple members of one house; we adopted a well-developed and validated lottery method which gives the same probability of each willing individual being recruited for the study. Students or the educated communities are more likely to understand research activities and show more willingness to participate in such surveys. Third, our sample consisted of 2/3 males whereas the census shows a higher percentage of females; this creates unequal distribution, and the sample may not be truely representative. This effect is due to the reluctance of females to participate, and also the lower education level of females, which is also a hindering factor in being part of an epidemiological study. This could be overcome by introducing female research assistants for interviewing females. Fourth, we selected only one individual from a house even if multiple members were willing. This effect reduced the repetition of the same responses, as observed in previous studies, since when we approach multiple members the answers are almost the same and sometimes they even ask us to copy the answers from another member’s questionnaire. Fifth, this study focused in Punjab only so cannot be generalized to the whole country. Nevertheless, as the first survey of its kind from the most populated region in Pakistan, the current study provided us a clearer picture of the KAP of common ocular conditions among the general public in Pakistan. Lastly, we were unable to check interviewer reliability. Our resources, time and population did not allow two interviewers to check inter-rater reliability (two interviewers interviewing the same individual). However, data collection was carried out by trained and diligent data collectors, and was cross-checked for completeness and thoroughness by the PI.

## 5. Conclusions

This study demonstrates the poor knowledge among the general community of Pakistan regarding the most common ocular diseases. Knowledge regarding preventive eye diseases like ARMD and glaucoma is quite low. Similarly, the attitude towards the eye checkup is low, and the better the education, the higher the rate of eye check. Therefore, there is a need to disseminate knowledge of the benefits of proper eye checks throughout the population. Furthermore, our study demonstrates that intensive improvements are needed in health literacy and public interventions, specifically in old age people, males and respondents of lower education.

## Figures and Tables

**Table 1:** Demographic illustration.

Variables		Number (*N*)	Percentage (%)
**Age (32.92 ± 11.4), years**			
	18–30	1134	56.2
	31–45	596	29.5
	46–60	242	12.0
	More than 60	47	2.3
**Gender**			
	Male	1309	64.8
	Female	710	35.2
**Residence**			
	Urban	980	48.5
	Rural	1039	51.5
**Education**			
	Nil	62	3.1
	1–5	420	20.8
	6–10	464	23.0
	10–12	333	16.5
	More than Intermediate	740	36.6
**Occupation**			
	Student	828	41.0
	Farmer (kisan)	276	13.7
	Laborer	184	9.1
	Housewife	218	10.8
	Teacher	194	9.6
	Business man	222	10.5
	Other	53	2.6
	None	43	2.1
**Socioeconomic Status (SES)**			
	Inadequate resources for complete year	222	11.0
	Inadequate for a few times	462	22.9
	Balanced	1122	55.6
	Adequate resources for most of the time	213	10.5

**Table 2:** Knowledge related items in terms of gender, age, education, locality and SES.

**Disease Related Questions**	**Gender**		**Age Group**				**Education Status**				
	**Male *n* = 1309 (%)**	**Female *n* = 710 (%)**	**18–30 *n* = 1134 (%)**	**31–45 *n* = 596 (%)**	**46–60 *n* = 242 (%)**	**>60 *n* = 47 (%)**	**Nil *n* = 62 (%)**	**1–5 *n* = 420 (%)**	**6–10 *n* = 464 (%)**	**10–12 *n* = 333 (%)**	**More Than Inter *n* = 740 (%)**
Red eye	738 (56.4)	493 (69.4)	758 (66.8)	307 (51.5)	133 (54.9)	33 (70.2)	24 (38.7)	198 (47.1)	254 (54.7)	222 (66.7)	533 (72.0)
Refractive error/Blurred vision	648 (49.5)	461 (64.9)	666 (58.8)	295 (49.5)	129 (53.3)	19 (40.4)	36 (58)	203 (48.3)	197 (42.4)	197 (59.1)	476 (64.3)
Cataract	674 (51.5)	470 (66.2)	707 (62.3)	298 (50.0)	116 (47.9)	23 (48.9)	30 (48.4)	190 (45.2)	203 (43.7)	226 (67.9)	495 (66.9)
Trachoma	438 (33.5)	368 (51.8)	565 (49.8)	167 (28.0)	64 (25.4)	10 (21.3)	17 (27.4)	91 (21.6)	128 (27.6)	166 (49.8)	404 (54.6)
Glaucoma	502 (38.3)	369 (52.0)	565 (49.8)	220 (36.9)	70 (28.9)	16 (34.0)	20 (32.2)	135 (32.1)	160 (34.5)	169 (36.4)	387 (52.3)
Pterygium	421 (32.2)	352 (49.6)	539 (47.5)	171 (28.7)	53 (21.9)	10 (21.3)	12 (19.3)	109 (25.9)	138 (29.7)	153 (33.0)	361 (48.8)
Age-related macular degradation.	362 (27.6)	273 (38.4)	416 (36.7)	164 (27.5)	45 (18.6)	10 (21.3)	4 (6.4)	105 (25.0)	99 (21.3)	121 (36.3)	306 (41.3)
Diabetic retinopathy	607 (46.4)	436 (61.4)	610 (53.8)	283 (47.5) *	129 (53.3) *	21 (44.7) *	20 (32.2)	179 (42.6)	215 (46.3)	186 (55.8)	443 (59.9)
Vision loss can be prevented	837 (63.9)	539 (75.9)	821 (72.4)	366 (61.4)	157 (64.8)	32 (68.1)	42 (67.7)	247 (58.8)	291 (62.7)	239 (71.8)	557 (75.3)
	**Residence**		**SES**								
	**Urban *n* = 980 (%)**	**Rural *n* = 1039 (%)**	**Inadequate Resources for Complete Year *n* = 222 (%)**		**Inadequate for a Few Times *n* = 462 (%)**		**Adequate Resources for Most of the Time *n* = 213 (%)**				
Red eye	534 (54.5)	697 (67.1)	117 (52.7)		205 (44.4)		137 (64.3)				
Refractive error/Blurred vision	517 (52.7) *	592 (57.0) *	122 (54.9)		205 (44.4)		120 (56.3)				
Cataract	583 (59.5)	561 (54)	116 (52.2) *		274 (59.3) *		123 (57.7) *				
Trachoma	410 (41.8) *	396 (38.1) *	100 (54.0)		159 (34.4)		103 (61.0)				
Glaucoma	422 (43.1) *	449 (43.2) *	112 (50.4)		184 (39.8)		114 (53.5)				
Pterygium	391 (39.9) *	382 (36.8) *	121 (54.5)		161 (34.8)		89 (41.8)				
Age-related macular degradation	329 (33.6)	306 (29.4)	96 (43.2)		151 (32.7)		67 (31.4)				
Diabetic retinopathy	485 (49.5) *	558 (53.7) *	106 (47.7)		212 (45.9)		122 (57.3)				
Vision loss can be prevented	642 (65.5)	734 (70.6)	140 (63.1)		261 (56.5)		154 (72.3)				

* Indicated the insignificant correlation between the variables and responses.

**Table 3:** Demographics of each eye-related problem (AOR, 95%CI Adjusted for Age, Gender, Locality, Education and SES).

Demographic Variable	Red Eye	Refractive Error	Cataract	Trachoma	Glaucoma	Pterygium	Age-related Macular Degradation	Diabetic Retinopathy	Vision Loss Can Be Prevented
**Age**									
18–30	1	1	1	1	1	1	1	1	1
31–45	0.48 (0.28, 0.97)	1.31 (0.70, 2.44)	0.92 (0.50, 1.70)	1.71 (0.82, 3.58)	1.20 (0.63, 2.28)	1.75 (0.88, 3.46)	1.06 (0.50, 2.23)	0.72 (0.42, 1.47)	0.82 (0.44, 1.66)
45–60	0.34 (0.17, 0.68)	01.24 (0.68, 2.36)	0.84 (0.46, 1.55)	1.09 (0.52, 2.28)	0.96 (0.50, 1.82)	1.09 (0.52, 2.27)	1.00 (0.48, 2.09)	0.88 (0.47, 1.62)	0.67 (0.35, 1.29)
More than 60	0.45 (0.22, 0.92)	1.51 (0.79, 2.88)	0.90 (0.47, 1.69)	1.21 (0.56, 2.61)	0.72 (0.37, 1.42)	0.88 (0.41, 1.91)	0,71 (0.32, 1.56)	1.34 (0.71, 2.53)	0.81 (0.41, 1.61)
**Gender**									
Male	1	1	1	1	1	1	1	1	1
Female	1.55 (1.25, 1.92)	1.69 (1.38, 2.07)	1.54 (1.25, 1.88)	1.68 (1.37, 2.07)	1.51 (1.23, 1.84)	1.83 (1.49,2.25)	1.42 (1.15, 1.75)	1.75 (1.43, 2.14)	1.63 (1.30, 2.03)
**Residence**									
Urban	1	1	1	1	1	1	1	1	1
Rural	2.08 (1.71, 2.55)	1.27 (1.05, 1.53)	0.90 (0.75, 1.09)	1.08 (0.89, 1.31)	1.19 (0.98, 1.43)	1.07 (0.88, 1.30)	0.98 (0.80, 1.20)	1.28 (1.06, 1.54)	1.37 (1.12, 1.67)
**Education**									
Nil	1	1	1	1	1	1	1	1	1
0–5	1.69 (0.91, 2.83)	0.70 (0.40, 1.22)	0.88(0.51, 1.51)	0.6 (0.36, 1.24)	0.99 (0.55, 1.77)	1.40 (0.71, 2.77)	4.78 (1.68, 13.58)	1.695 (0.95, 3.01)	0.72 (0.40, 1.29)
6–10	2.54 (1.45, 4.51)	0.61 (0.35, 1.07)	0.85 (0.49, 1.46)	1.00 (0.54, 1.85)	1.23 (0.69, 2.19)	2.00 (1.01, 3.94)	4.32 (1.51, 12.33)	2.30 (1.28, 4.10)	0.93 (0.52, 1.68)
10–12	3.94 (2.16, 7.18)	1.04 (0.58, 1.85)	2.09 (1.18, 3.70)	1.90 (1.02, 3.55)	1.82 (1.00, 3.31)	2.85 (1.42, 5.71)	7.40 (2.58, 21.21)	3.03 (1.67, 5.53)	1.23 (0.66, 2.27)
More than Intermediate	4.66 (2.60, 8.33)	1.24 (0.71, 2.16)	2.07 (1.19, 3.60)	2.29 (1.25, 4.21)	1.98 (1.11, 3.54)	3.34 (1.69, 6.58)	9.79 (3.45, 27.80)	3.64 (2.03, 6.52)	1.36 (0.75, 2.47)
**SES**									
Insufficient funds for whole year	1	1	1	1	1	1	1	1	1
Insufficient for some time	0.890 (0.59, 1.33)	1.20 (0.81, 1.78)	1.11 (0.74, 1.64)	1.31 (0.87, 1.97)	1.16 (0.78, 1.72)	2.47 (1.64, 3.70)	2.13 (1.52, 3.49)	0.91 (0.61, 1.35)	0.83 (0.54, 1.26)
Balanced	0.63 (0.44, 0.90)	0.82 (0.58, 1.16)	1.55 (1.09, 2.20)	0.85 (0.59, 1.21)	0.76 (0.54, 1.08)	1.08 (0.76, 1.54)	1.50 (1.04, 2.17)	0.85 (0.60, 1.20)	0.64 (0.44, 0.92)
Sufficient funds for most of times	1.53 (1.10, 2.11)	1.32 (0.97, 1.80)	1.18 (0.87, 1.61)	0.86 (0.63, 1.18)	0.69 (0.51, 0.94)	0.94 (0.68, 1.28)	1.03 (0.74 1.43)	1.03 (0.75, 1.39)	1.21 (0.86, 1.69)

**Table 4:** Demographic association with the total knowledge score by Rasch analysis.

Variable	Un Adjusted β (95% CI)	*p*	Adjusted β (95% CI)	*p*
Age	−0.02 (−0.03, −0.02)	<0.001	−0.008 (−0.01, −0.002)	0.010
Gender	0.72 (0.59, 0.86)	<0.001	0.53 (0.39, 0.66)	<0.001
Residence	0.03 (−0.09, 0.16)	0.586	0.22 (0.09, 0.34)	0.001
Education	0.37 (0.32, 0.42)	<0.001	0.31 (0.25, 0.37)	<0.001
SES	0.09 (0,01, 0.17)	0.030	−0.04 (−0.11, 0.03)	0.297

**Table 5:** Demographic relationship with attitude of general population (“Will you seek eye treatment for the disease?”).

Variable		No		Yes		AOR (95% CI)	*p*
	*N*	*N*	%	N	%		
**Age, years**							
18–30	1134	179	15.8	955	84.2	1	
31–45	596	136	22.8	460	77.3	1.47 (0.72, 3.09)	0.032
46–60	242	37	15.3	205	84.7	1.06 (0.59, 2.17)	0.227
More than 60	47	11	23.4	36	76.6	1.69 (0.78, 3.66)	0.868
**Gender**							
Male	1309	243	18.5	1066	81.5	1	
Female	710	120	16.9	590	83.1	0.98 (0.75, 1.27)	0.879
**Residence**							
Urban	980	185	18.9	795	81.1	1	
Rural	1039	178	17.1	861	82.9	1.21 (0.95, 1.54)	0.109
**Education**							
Nil	62	4	6.4	58	93.6	1	
1–5	420	94	22.4	326	77.6	0.25 (0.08, 0.72)	<0.001
6–10	464	106	22.8	358	77.2	0.24 (0.08, 0.70)	0.010
10–12	333	62	18.6	271	81.4	0.32 (0.11, 0.93)	0.009
More than Intermediate	740	97	13.1	643	86.9	0.46 (0.16, 1.33)	0.152
**SES**							
Inadequate resources for complete year	222	55	24.8	167	75.2	1	
Inadequate for a few times	462	88	19.0	374	81.0	1.02 (0.64, 1.61)	0.925
Balanced	1122	171	15.2	951	84.8	1.58 (1.05, 2.39)	0.027
Adequate resources for most of the time	213	49	23.0	164	77.0	1.82 (1.26, 2.62)	0.001

**Table 6:** Association of demographic characters with practice regarding eye check.

Variable	N	More Than Once in a Year	At Least Once in a Year	When You Think You Have Eye Disease	Never	*p*
		*N* (%)	*N* (%)	N (%)	N (%)	
**Age, years**						
18–30	1134	241 (21.2)	270 (23.8)	375 (33.2)	248 (21.8)	<0.001
31–45	596	72 (12.1)	136 (22.0)	245 (41.1)	143 (23.9)	
46–60	242	30 (12.4)	19 (7.8)	111 (45.9)	82 (33.9)	
More than 60	47	4 (8.5)	10 (21.3)	22 (46.8)	11 (23.4)	
**Gender**						
Male	1309	207 (15.8)	235 (18.0)	527 (40.2)	340 (26.0)	<0.001
Female	710	140 (19.7)	200 (28.2)	226 (31.8)	144 (20.3)	
**Residence**						
Urban	980	191 (19.5)	233 (23.8)	375 (38.2)	181 (18.5)	<0.001
Rural	1039	156 (15.0)	202 (19.4)	378 (36.4)	303 (29.2)	
**Education**						
Nil	62	11 (17.7)	2 (3.2)	32 (51.7)	17 (27.4)	<0.001
1–5	420	75 (17.9)	67 (15.9)	158 (37.6)	120 (28.6)	
6–10	464	38 (8.2)	135 (29.1)	167 (36.0)	124 (26.7)	
10–12	333	56 (16.8)	69 (20.8)	134 (40.2)	74 (22.2)	
More than Intermediate	740	167 (22.5)	162 (21.9)	262 (35.5)	149 (20.1)	
**SES**						
Inadequate resources for complete year	222	73 (32.9)	26 (11.7)	80 (36.0)	43 (19.4)	<0.001
Inadequate for a few times	462	71 (15.4)	133 (28.8)	183 (36.9)	75 (16.2)	
Balanced	1122	159 (14.2)	212 (18.9)	417 (37.2)	334 (29.7)	
Adequate resources for most of the time	213	44 (20.6)	64 (30.0)	73 (34.4)	32 (15.0)	
